# Risk of complications after core needle biopsy in pheochromocytoma/paraganglioma

**DOI:** 10.1530/ERC-22-0354

**Published:** 2023-06-02

**Authors:** Liang Zhang, Tobias Åkerström, Kazhan Mollazadegan, Felix Beuschlein, Karel Pacak, Britt Skogseid, Joakim Crona

**Affiliations:** 1 Department of Medical Sciences, Uppsala University, Uppsala, Sweden; 2 Department of Surgical Sciences, Uppsala University, Uppsala, Sweden; 3 Klinik für Endokrinologie, Diabetologie und Klinische Ernährung, Universitätsspital Zürich (USZ) and Univeristät Zürich (UZH), Zurich, Switzerland; 4 Medizinische Klinik und Poliklinik IV, Klinikum der Universität München, Munich, Germany; 5 Section on Medical Neuroendocrinology, Eunice Kennedy Shriver National Institute of Child Health and Human Development, National Institutes of Health, Bethesda, Maryland, USA

**Keywords:** pheochromocytoma, paraganglioma, core needle biopsy, diagnosis, risk

## Abstract

Core needle biopsy (CNB) has been used with caution in pheochromocytoma and paraganglioma (PPGL) due to concerns about catecholamine-related complications. While it is unclear what scientific evidence supports this claim, it has limited the acquisition of biological samples for diagnostic purposes and research, especially in metastatic PPGL. We performed a systematic review and individual patient meta-analysis to evaluate the risk of complications after CNB in PPGL patients. The primary and secondary objectives were to investigate the risk of death and the occurrence of complications requiring intervention or hospitalization, respectively. Fifty-six articles describing 86 PPGL patients undergoing CNB were included. Of the patients (24/71), 34% had metastases and 53.4% (31/58) had catecholamine-related symptoms before CNB. Of the patients (14/41), 34.1% had catecholamine excess testing prior to the biopsy. No CNB-related deaths were reported. Four patients (14.8%, 4/27) experienced CNB-related complications requiring hospitalization or intervention. One case had a temporary duodenal obstruction caused by hematoma, two cases had myocardial infarction, and one case had Takotsubo cardiomyopathy. Eight patients (32%, 8/25) had CNB-related catecholamine symptoms, mainly transient hypertension, excessive diaphoresis, tachycardia, or hypertensive crisis. The scientific literature does not allow us to make any firm conclusion on the safety of CNB in PPGL. However, it is reasonable to argue that CNB could be conducted after thorough consideration, preparation, and with close follow-up for PPGL patients with a strong clinical indication for such investigation.

## Introduction

Pheochromocytomas (PCCs) and paragangliomas (PGLs, together denoted as PPGLs) are rare endocrine tumors originating from chromaffin cells in the adrenal medulla (PCC) and extra-adrenal paraganglia (PGL). Sympathetic PPGLs secrete excess catecholamines that result in an increased risk of cardiovascular disease (Mete *et al.* 2022, Nölting *et al.* 2022). In a retrospective case–control study including 109 PCCs, the frequency of cardiovascular events was about 14%, considerably higher than in a matched population without PPGLs (Stolk *et al.* 2013). In addition to this cardiovascular morbidity, metastatic disease affects up to 25% of PPGL patients and is the main cause of mortality (Jimenez *et al.* 2022). In fact, metastatic PPGL can only be cured in a minority of patients that can undergo surgical resection. Nevertheless, for metastatic PPGL, surgery is mostly used for debulking purposes and systemic therapies mostly offer disease stabilization (Crona *et al.* 2017, Fishbein *et al.* 2021). While core needle biopsy (CNB)-based disease characterization is now facilitating individualized anti-cancer therapy strategies for many tumor entities, lacking effective therapy is the current therapeutic dilemma in metastatic PPGL. This situation is rather unique for PPGL where fear that biopsy could lead to catecholamine-related complications may halter the implementation of individualized therapy. Surgical resection is the cornerstone of PPGL treatment. By using minimal invasive techniques supported by modern anesthesia, such procedures are considered safe even in patients with catecholamine-related symptoms and signs (Neumann *et al.* 2019, Fu *et al.* 2020, Li *et al.* 2020).

For metastatic cancer patients that are not eligible for surgery, CNB is routinely used to obtain tissue samples for diagnostics and research. This procedure is generally considered to be safe for cancer patients, with only a small minority requiring hospitalization or intervention (Bravo *et al.* 2001, Rockey *et al.* 2009). For patients with metastatic PPGL, CNB may provide a pathology-confirmed diagnosis as well as enable the study of exploratory questions related to tumor biology (Fishbein *et al.* 2021). It is also clear that metastatic PPGL lesions often present with a different behavior as compared to its primary tumor. Such differences in biology may indeed prove to be clinically relevant and could be translated into new therapeutic possibilities through genetic guided therapy or emerging immunotherapies (Zethoven *et al.* 2022, Ghosal *et al.* 2023). But until now, such analyses of the tumor biology of metastases lesions through CNB have been avoided due to fear that tumor manipulation may cause a release of catecholamines leading to patient complications. We hypothesized that such considerations were based on anecdotal evidence and that CNB should be considered as having low risk in patients with metastatic PPGL. This systematic review and meta-analysis aims to clarify the risk of CNB-related complications in the PPGL population.

## Materials and methods

This study followed the Preferred Reporting Items for Systematic Reviews and Meta-Analyses (PRISMA) guidelines (Hutton *et al.* 2015), and all objectives were predefined under the study protocol (Supplementary Appendix, see section on supplementary materials given at the end of this article). The primary objective was to establish the risk of death after CNB. The secondary objective was to describe the occurrence of serious complications requiring hospitalization or intervention.

### Eligibility criteria

Studies fulfilling the following criteria were included: original articles or case reports describing percutaneous CNB of localized or metastatic PPGL. Review publications as well as reports only describing patients with head and neck PGLs or patients subjected to fine needle aspiration were excluded. In the scenario where authors published multiple reports, with a potential for patient overlap, the most recent publication was selected.

### Search strategy, study selection, and data extraction

Two investigators performed a systematic PUBMED (https://pubmed.ncbi.nlm.nih.gov) search to identify relevant reports. A secondary literature search was performed using Google Scholar (https://scholar.google.com) as well as the reference lists of the already identified reports. We selected original reports published until 30 June 2022 and used the search terms ‘pheochromocytoma’ OR ‘paraganglioma’ AND ‘biopsy’ to identify relevant articles to be screened for inclusion. Only publications in English language were considered. Reports were initially screened by title for relevance, and potentially interesting articles had its abstract reviewed. All articles were selected after screening the full text if available. Disagreements were resolved by discussion and ultimately by the decision of the senior author (JC). LZ and JC independently accessed all cases and extracted data items (defined in Supplementary Appendix). Items not explicitly reported were noted as not reported.

### Risk of bias assessment

The risk of bias was assessed by two investigators (LZ and JC), and disagreements were decided by the senior author (JC). Each study was assessed using a modified Newcastle–Ottawa Scale (Wells *et al.* 2011, Crona *et al.* 2019) that had been adopted to evaluate the following: availability of PPGL patient characteristics, cohort selection strategy, and reporting of outcome data (Supplementary Appendix).

### Analysis plan

The threshold to perform statistical analyses had been predefined to 48 cases. With an anticipated background risk of death after biopsy of 0.01% in general diseases, 48 patients would be needed to identify a 2% mortality risk with alpha 0.05, beta 0.2, and power 0.8 (https://clincalc.com/stats/samplesize.aspx). While a 2% mortality risk would be clinically unacceptable, this number was empirically selected as having adequate statistical power while being realistic to identify in the literature. Categorical variables were presented as numbers and percentages. Continuous normally distributed data were presented as mean ± s.d. and non-normally distributed data were presented as median (range).

## Results

### Study selection and characteristics

**Figure 1:**
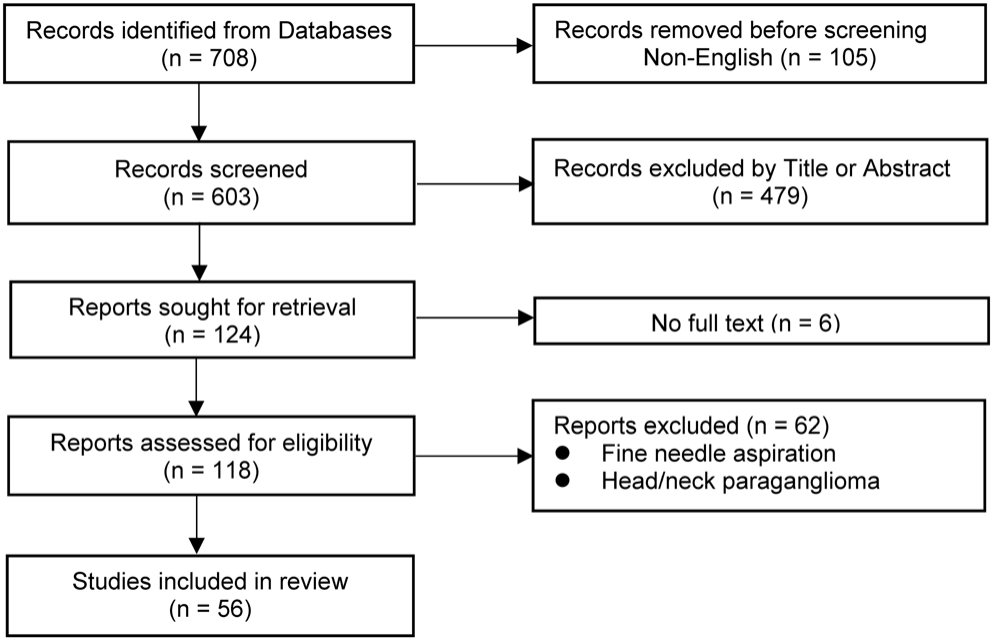
Flowchart of study selection. Flowchart describing study selection. Out of 708 initial findings by literature search, 56 studies fulfilled criteria and were included.

The initial search found 708 unique records that were subjected to screening of title and its abstract (Fig. 1). Among these, 105 articles were excluded as they were not in English. Further, 479 papers were excluded after scanning the title and abstract. A total of 124 studies were selected for full-text assessment, though 6 of them were not available. Of these 118 reviewed papers, 62 studies were excluded as they described fine needle aspiration and/or head and neck PGLs. Finally, a total of 56 studies were included in the present analysis. This included 11 case series and 45 case reports (fully described in the Supplementary Appendix). Studies were conducted in Europe (*n* = 9), Asia (*n* = 25), and North America (*n* = 22).

### Risk of bias assessment

**Figure 2:**
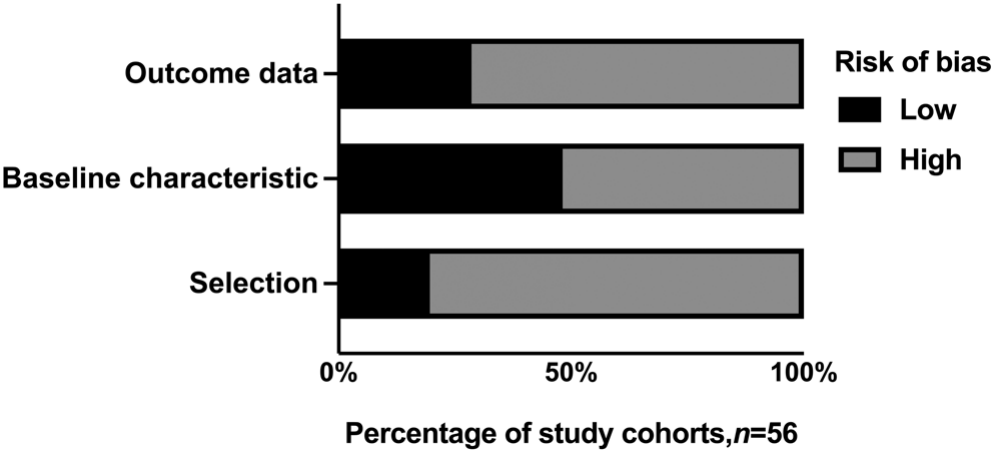
Bias assessment. Bar chart describing the outcome from study bias assessment. All studies were categorized as having low or high risk of bias accordingly to three different categories: study selection, availability of pheochromocytoma, and paraganglioma patient characteristics as well as reporting of outcome data.

Out of the 56 included studies, 45 were case reports and were thus considered to be at high risk of selection bias (Fig. 2). A high risk of bias was also noted in 29 of the articles that lacked information to perform a satisfactory description of PPGL patient characteristics. Finally, in 40 of the included studies, there was a high risk of bias due to unsatisfactory description of patient outcomes.

### Study cohort and baseline characteristics

A total of 56 articles with 86 PPGL patients undergoing CNB were included (Table 1). There were 31 male and 24 female patients, and the median age was 53 (range 10–76) years. Mean primary tumor size was 7.3 ± 5.2 cm with a total of 34% (24/71) of patients having metastases. Catecholamine-related symptoms before CNB were reported in 53.4% (31/58) of cases.

**Table 1:** Baseline PPGL patient characteristics.

Characteristic	Values
Total *n*	86
PPGL primary tumor size, cm	7.3 ± 5.2
Gender	
		Female	24
		Male	31
NR	31
Age, years (median (range))	53 (10–76)
Genetics	
		*RET*	1
*VHL*	1
*NF1*	1
*SDHB*	2
NR	81
Metastatic disease before biopsy	
		Confirmed or suspected	24
		None	47
NR	15
Catecholamine-related symptom	
		With symptoms	31
		None	27
NR	28
Testing of catecholamine excess	
Normal	13
Elevated only	11
Mildly elevated	3
Markedly elevated	14
Avoided	2
Not reported	43
Biochemical testing time	
Prior to biopsy	14
After biopsy	27
Not reported	45
Preoperative adrenoceptor blockade	
	With alpha-blockade	4
	None	45
NR	36

NR, not reported; PPGL, pheochromocytoma/paraganglioma.

Catecholamine excess was defined as elevated plasma or urine catecholamine or catecholamine metabolite levels. Tests of catecholamine excess were performed in 41 cases, of which 13 (31.7%) were reported at normal levels, 11 (26.8%) suggested elevations but no values were reported, 3 (7.3%) showed mildly elevated, and 14 (34.2%) showed markedly elevated results. In addition, we noted that 14 (34.2%) out of 41 patients, with tests of catecholamines excess, had their testing performed prior to CNB, while in 27 cases (65.8%), this was done after CNB. Alpha-adrenoceptor blockade had been administrated before CNB in 8% (4/50) of patients.

### Outcomes

Zero (0%, 0/86) CNB-related deaths were reported. Four patients (14.8%, 4/27) experienced CNB-related complications requiring hospitalization or intervention. None of these four patients had PPGL suspected before CNB and were not reported to have received monitoring or preparation as recommended for PPGL (Table 2). Among all patients, CNB-related catecholamine symptoms and signs were reported in eight cases (32%, 8/25), including transient hypertension, chest pain, excessive diaphoresis, and tachycardia (Table 2) (Lynn *et al.* 1987, Takezawa *et al.* 2001, Hassan *et al.* 2003, Dalal *et al.* 2005, Sood *et al.* 2007, Cai *et al.* 2014, Meydan *et al.* 2016). Among these eight patients, one received alpha-blockade prior to CNB. We did not identify any patients who had their PPGL confirmed prior to CNB and who experienced complications. Further, no case of CNB-related tumor seeding was documented.

**Table 2:** Outcome of core needle biopsy in PPGL patients.

Variables	Case series (*n* = 39)	Case reports (*n* = 47)
CNB-related deaths	0	0
CNB-related complications	1 positive	3 positive
	14 none	9 none
	24 NR	35 NR
CNB-related catecholamine symptom	Lynn *et al.* 1987: Two cases with markedly hypertensive in the postoperative period, with systolic blood pressures of over 200 mm Hg.	Takezawa *et al.* 2001: Hypertensive crisis
		Hassan *et al.* 2003: Blood pressures increased to 217/110 mmHg	Dalal *et al.* 2005: Hypertensive and tachycardia	Sood *et al.* 2007: Crushing chest pain	Cai *et al.* 2014: Blood pressures suddenly rose to 160/100 mmHg and then came back to 130/90 mmHg	Meydan *et al.* 2016: Chest pain, palpitations, and excessive diaphoresis

CNB, core needle biopsy; NR, not reported; PPGL, pheochromocytoma/paraganglioma.

### Descriptive analysis of patients with complications requiring hospitalization or intervention

Patient characteristics and outcomes are summarized in Table 3. The first patient was a female aged 24 years old (Hassan *et al.* 2003). She had a vaginal mass of increasing size for 2 years and showed no catecholamine-related symptoms. Therefore, no testing of catecholamine excess had been performed before CNB. An episode of acute pulmonary edema followed CNB with sharply increased blood pressure up to 217/110 mmHg. The authors diagnosed a myocardial infarction with an elevated troponin I level of 3 ng/mL (normal 0.0–1.4 ng/mL) and an echocardiogram showing an ejection fraction of 35%. During the 2 weeks of hospitalization, the patient developed a re-entry tachycardia that was ablated as well as deep venous thrombosis. The mass, which was confirmed as PGL, was surgically removed following alpha-adrenoceptor blockade preparation.

**Table 3:** Characteristics of patients with core needle biopsy-related complications.

No.	Sex/age	Tumor location/size	Test of catecholamine excess	Catecholamine-related symptoms	Core needle biopsy-related complications
P1 (Hassan *et al.* 2003)	Female, 24	Vaginal, 3 cm	NR	No	Acute pulmonary edema, myocardial infarction
P2 (Sood *et al.* 2007)		Male, 74			Para-aortic, 7 cm	Elevated after biopsy	No	Myocardial infarction
P3 (Goers *et al.* 2013)	NR		NR	NR	NR				Hematoma, temporary duodenal obstruction
P4 (Meydan *et al.* 2016)		Male, 49				Retroperitoneal, 6.4 cm	Elevated after biopsy	No		Takotsubo cardiomyopathy

NR, not reported; PPGL, pheochromocytoma/paraganglioma.

The second patient was a 74-year-old man with a history of prostate cancer, who had presented with altered bowel habits and weight loss (Sood *et al.* 2007). The abdominal CT scan detected a 7 cm left para-aortic mass with a preliminary diagnosis before biopsy of a soft tissue sarcoma. Crushing chest pain occurred within minutes of the CNB, which was diagnosed as a myocardial infarction. Catecholamine measurement was not conducted. A second biopsy confirmed a PGL 6 months later, and by then, catecholamine excess was reported.

The third patient had a hematoma after CNB, resulting in temporary duodenal obstruction (Goers *et al.* 2013). Due to an inflammatory reaction around the lesion, the patient had to undergo a conversion to open surgery during laparoscopic surgery. No further patient characteristic was reported.

The fourth case was a 49-year-old man with a weight loss of 10 kg over the past year and poorly defined complaints of anxiety (Meydan *et al.* 2016). A 6 cm retroperitoneal mass was discovered, and no diagnosis of PPGL was considered due to a lack of typical clinical hallmarks (hypertension, headaches, and diaphoresis). CNB was conducted by an experienced interventional radiology team, and vital signs were stable during the procedure. However, the patient experienced chest pain, palpitations, and excessive diaphoresis 1 h after the biopsy and was then diagnosed with Takotsubo cardiomyopathy. Electrocardiogram indicated ST segment elevations suggesting a diagnosis of myocardial infarction. However, angiography examination showed no signs of coronary artery abnormality while revealing marked left ventricular dysfunction with an estimated ejection fraction of 35%. These complications resolved within 12 h. The tissue sample supported the diagnosis of a retroperitoneal PGL, and subsequent catecholamine excess testing was positive.

## Discussion

We have performed a systematic review and individual meta-analysis to evaluate the risk of CNB-related complications in PPGL patients. To our knowledge, this is the first review and analysis on this topic. There were zero deaths related to CNB. But due to a high degree of bias, the relationship between complications and PPGL characteristics remained difficult to assess.

### Study limitations

Our review and analysis have several limitations. First, the reviewed articles consisted of mostly case reports and a few small case series. This could be explained by the rare nature of PPGL as well as the hesitation to perform any invasive procedures in this patient group. Furthermore, the eligibility criteria may have excluded some special cases with catecholamine-secreting parasympathetic tumors (5% of head and neck PGLs can secrete catecholamines) and patients subjected to fine needle aspiration. We acknowledge the potentially significant limitation of not including articles on fine needle aspiration in the literature review since this intervention is similar to CNB and is expected to have an overlapping spectrum of complications. Thus, we consider that the study population had a high risk of selection bias. Both under- and over-reporting of PPGL patients having CNB complications can be argued for. Second, missing data also cause a high risk of bias and did not allow us to perform any statistical comparisons to identify potential risk factors for CNB complications. Most notably, information on catecholamine excess testing, an important predictor for cardiovascular events, was often missing from the records of many articles included in this study. In addition, laboratory workup sometimes consisted of analyses with lower sensitivity for PPGL diagnosis, including vanillylmandelic acid and urinary catecholamines. This could potentially explain why 13/41 (31.7%) had normal results of catecholamine excess testing and only 4 patients were treated with alpha-adrenoceptor blockade prior to biopsy. It was thus difficult to generate any hypotheses on the impact and relationship of catecholamines in the four patients that experienced complications requiring hospitalization or intervention. Finally, for outcome reporting, our analysis was based on the assumption that patients did not die if follow-up information was lacking.

Taking into account these reasons, an independent statistician was consulted, and it was decided to present the main results descriptively without calculating 95% confidence intervals as originally planned in the study protocol.

### Interpretation of results

When a PPGL patient is considered for CNB, both the clinical impact and the risk of the intervention must be considered. This study aimed to provide data to more precisely determine the risk of complications, and we conclude that there was no evidence for CNB-related deaths in PPGL patients. This is even as only four patients had documented treatment with alpha-adrenoceptor blockade, which is recommended for 7–14 days before biopsy procedures (Fishbein *et al.* 2021). For patients with PPGL, tumor manipulation can evidently result in CNB-related catecholamine symptoms and complications. But in included research, we failed to identify any patients with prior confirmed PPGL that had CNB-related complications (Yashiro *et al.* 2005, Kim *et al.* 2014). Thus, in a well-selected and prepared population of PPGLs, it is reasonable to argue that the risk of complications is limited, in line with the minimal morbidity and mortality after surgery (Araujo-Castro *et al.* 2021, Fagundes & Almeida 2022). As a minimally invasive procedure, one could even speculate that CNB should be associated with a lower complication rate compared to surgery.

Despite performing this systematic review, we were unable to draw any firm conclusions on the available direct evidence describing the safety of CNB in patients with PPGL. Our data should therefore be interpreted in the wider context of the scientific literature available on the safety of interventions in PPGL, including fine needle aspiration. Although considered less invasive than CNB, there is one case with bleeding and death (McCorkell & Niles 1985) after fine needle aspiration described in PCC. There are also reports on serious catecholamine-related cardiovascular complications after fine needle aspiration of PPGL (Lambert *et al.* 1985, Casola *et al.* 1986, Quayle *et al.* 2007, Mamlouk *et al.* 2009, Vanderveen *et al.* 2009, Delivanis *et al.* 2016). On the other hand, it is well demonstrated that in modern healthcare surgery can be performed on PPGL patients without causing any relevant complications (Neumann *et al.* 2019, Fu *et al.* 2020, Li *et al.* 2020). How to balance this information into the decision-making for individual patients is a real challenge.

Our interpretation is that CNB should be considered for a minority of PPGL patients and probably avoided in those with uncontrolled cardiovascular symptoms and/or with a high cardiovascular risk score. While in metastatic PPGL patients without cardiovascular risk factors, having no or limited symptoms of catecholamine secretion, there is no direct evidence to suggest that CNB is dangerous. Still, due to the discussed uncertainties, it is recommended to have such patients under close observation.

## Future recommendations

This work clearly identified a gap in evidence and pinpointed what scientific data would be useful to improve the care of PPGL patients, especially in those with metastases. There is clearly a lack of data in the literature to determine the risk of CNB in PPGL patients with satisfactory confidence. As such, we recommend further studies on this topic and that CNB should be performed with caution in PPGL patients until further evidence is available. While prospective studies will take a long time and require considerable resources, a retrospective study including all PPGL patients undergoing CNB at multiple institutions would probably provide more information on this topic. Research on this topic should be motivated by the potential of CNB to provide more detailed information on disease characteristics that could ultimately help to develop more effective treatment protocols for metastatic PPGL. What’s more, the fact that no patient with prior confirmed PPGL had CNB-related complications emphasizes the importance of raising awareness of PPGL in the medical community. Comprehensive medical history and clinical examination should be able to identify patients with potential PPGL to be subjected to catecholamine excess testing (Lenders *et al.* 2020).

## Conclusions

This study found zero deaths, but a minority of PPGL patients had catecholamine complications after CNB. The scientific literature does not allow to make any firm conclusion on the safety of CNB in PPGL. However, it is reasonable to argue that CNB may be a justifiable approach for investigating PPGL in patients with a strong clinical indication, provided that it is conducted with thorough consideration, adequate preparation, and close follow-up.

## Supplementary Materials

Supplementary Appendix

Supplementary Material

## Declaration of interest

The authors declare that there is no conflict of interest that could be perceived as prejudicing the impartiality of the research reported.

## Funding

L Zhang is funded by China Scholarship Councilhttp://dx.doi.org/10.13039/501100004543 (No.202106370081). J Crona is funded by Cancerfondenhttp://dx.doi.org/10.13039/501100002794. The study has been supported by Cancerfondenhttp://dx.doi.org/10.13039/501100002794 and Lions Cancerforskningsfond i Uppsala.
