# Supplementary material for: Risk of complications after core needle biopsy in pheochromocytoma/paraganglioma
Source: Endocr Relat Cancer. 2023 Jun 2;30(7):e220354. doi: 10.1530/ERC-22-0354 (PMC10304911; doi:10.1530/ERC-22-0354)
Supplement: Supplementary Appendix [file supplementary_table_1.pdf]

Supplementary appendix- Study Protocol

Sections 3. Study protocol dated 2022-05-30

Project plan

Systematic review and individual patient meta-analysis on the safety of tumor biopsy in patients with pheochromocytoma (PCC) and paraganglioma (PGL; collectively denoted as PPGL)

Background

Catecholamine excess, local growth, and metastatic disease all contribute to increased morbidity and mortality in PPGL patients[1-8]. Those with sympathetic PPGLs have an almost 10-fold higher incidence of cardiovascular events prior to their diagnosis[8]. Indeed, data from physiological experiments suggest detrimental effects on the cardiovascular system by catecholamines that may result in hypertension, tachyarrhythmias, and cardiac failure[8, 9]. Stolk *et al*., conducted a retrospective case-control study including 109 PCCs and 183 patients with essential hypertension, reported more frequent adverse cardiovascular events in PCC patients: 13.8% (95%CI 7.9-21.6%), compared to matched controls: 1.1% (95%CI 0.1-3.9%). The adverse events in the PCC group included seven cardiac infarctions, five patients with stroke/transient ischemic attacks and four cases of angina pectoris[8]. Anaesthesiologists reported high morbidity and mortality associated with PPGL, including hemodynamic crisis and organ failure upon diagnosis (also denoted as catecholamine crisis)[10]. Among 34 patients with catecholamine crisis that were admitted to the intensive care unit, the 90-day mortality was 27% (*n*=9/34)[11]. Among these 34 patients with PPGLs, 32 were not diagnosed at admission and were, therefore, not treated with pharmacological blockade. Data from two retrospective studies suggest that the risk of cardiovascular events is normalized or decreased after radical surgery[1, 2]. The cardiovascular consequences of a sympathetic PGL is estimated to be similar to a PCC.

Adrenal biopsy has been widely used on primary or metastatic adrenal tumors so as to distinguish malignancy. A meta-analysis described that adrenal biopsy has an overall 87% sensitivity and 100% specificity for diagnosis of malignancy. However, the safety of adrenal biopsy has not been clearly described due to limited data in previous literatures[12].In patients with PPGLs, tumor biopsy is recommended to be avoided due to fear of catecholamine-related adverse events caused by sudden catecholamine release upon tumor manipulation, however, the safety of biopsy remains unclear because of limited cases .Actually, biopsy is to some extent very important for mPPGL(metastatic PPGL) patient management, such as investigating the somatic mutation[13].The US Endocrine Society Clinical Practice Guidelines on PPGL recommend that all patients with hormonally functional tumors should undergo preoperative adrenoceptor blockade to prevent perioperative cardiovascular complications[14].The North American Neuroendocrine Tumor Society Consensus

Guidelines also recommends α-blockade to be used before biopsy [13]. Although clinically very important, both these recommendations are still based on low quality evidence. Previously published articles mostly focused on surgical related complications rather than cardiovascular events. A meta-analysis comparing laparoscopic transperitoneal and retroperitoneal adrenalectomy in PCC included data on 1893 interventions[15].Major complications were documented in 1-2% of cases and the mortality rate was 0%. Cardiovascular disease as a complication was grouped into the major complications group (consisting mainly of surgical complications) and was not analysed independently. The safety evaluation of PPGL tumor biopsy remains to be further studied.

Tumor biopsy is thought to be a safe procedure in patients without PPGL. In the setting of non-focal liver disease where a cutting needle approach was used, severe bleeding, that was classified as bleeding requiring hospitalization or intervention, occurred in about 1:2500-10000 biopsies[16, 17]. The mortality rate was less than or equal to 1:10000 liver biopsies. In the Copenhagen Prospective Personalized Oncology trial 500 patients underwent core needle biopsy of liver (n=271), lymph nodes (n=68), subcutaneous metastasis (n=36) and lung metastasis (n=25)[18]. Biopsy complications were observed in 15 patients including hematoma (n=6), pneumothorax (n=3) and others (n=6). A short hospitalization was required in 7 patients (median 1 day, range 1-4 days). No biopsy related deaths occurred.

PPGLs are rare tumors often with low evidence supporting some recommendations of current clinical practice. Those patients with mPPGL have an incurable disease that is complicated by symptoms from metastatic spread and hormonal overproduction. New and effective treatment alternatives could influence the natural course of this disorder, but requires a deeper understanding of disease biology. In particular, the processes underlying metastatic dissemination, treatment sensitivity and resistance, as well as disease evolution still evade our understanding. In order to study metastatic PPGL, biomaterial from such tumors is needed. Sampling of metastatic PPGL is avoided due to the feared risk of catecholamine release after such intervention. We believe that long-term consequences transfer into a slower pace of research and translating some data to the PPGL patient population.

Aim / objectives

To conduct a systematic review on the safety of biopsy in patients with PPGLs. If the material meets a pre-specified quality threshold we will perform a individual patient meta-analysis to study the risk of death in the overall population and the influence of potential risk factors to forecast death.

Method

This study followed the Preferred Reporting Items for Systematic Reviews and Meta-Analyses (PRISMA) workflow[19]. The study reviewed and analyzed published data.

Search strategy and selection process

Two investigators performed a systematic PUBMED searches to identify relevant reports. Upon completion, a secondary literature search using Google Scholar as well as reference lists of the previously identified reports were performed. We selected reports published until 30th June of 2022. We used the search terms "pheochromocytoma" OR "paraganglioma" AND "biopsy" to identify relevant articles to be screened for inclusion. Only publications in English language were considered. Reports were initially screened by title for relevancy and potentially interesting articles had its abstract reviewed to select potentially interesting articles to be screened in full text. Disagreements were resolved by decision of the senior author (JC).

Eligibility criteria

Studies fulfilling the following criteria were included: original articles or case reports describing percutaneous needle biopsy of localized or metastatic PCC or PGL. Review studies as well as reports only describing patients with head neck PGL or patients subjected to fine needle aspiration were excluded. In the scenario with authors publishing multiple reports with the potential of patient overlap, the most recent publication was selected.

Data collection process and data items

Data items:

Data items were extracted from each report separately using criteria as described below. Data items not clearly described were noted as "not available".

Table 1. Patient characteristics at the time of biopsy procedure

**Data item**	**Description**
PPGL primary tumour size	Continuous variable, cm
Gender	Female/male
Age	Years
Genetic syndrome	Yes/no, only negative if said that genetic testing was negative
Metastatic	Yes/no
Recurrent	Yes/no
Catecholamine excess	Defined as elevated catecholamine or catecholamine metabolite levels either in blood or urine and noted as following: 1. Normal; 2. Elevated but no recorded; 3. Mildly elevated (Elevated but less than 2.5-fold the upper limit);4. Markedly elevated (> 2.5-fold the upper limit);5. Not reported.
Catecholamine related symptoms and	Yes/no, defined as the presence of

signs	hypertension, palpitations, headache, or ongoing acute cardiovascular disease including stroke, heart failure, myocardial infarction etc
Preoperative adrenoceptor blockade	Yes/no

Table 2. Outcome measures

**Outcome measure**	**Definition**
Death related to biopsy procedure	Yes/no, if data on death is not reported it is assumed that patient did not die
Complications related to the biopsy procedure requiring hospitalization or intervention	Yes/no

In addition, the side effect profile after biopsy procedure will be studied qualitatively to describe any catecholamine related-adverse events.

<u>Quality and risk of bias assessment</u>
Risk of bias was assessed by two investigators (LZ, JC) and re-evaluation was conducted once any differences. The quality of each study will be assessed using the Newcastle-Ottawa Scale (NOS). Detailed criteria are available in the supplemental document.

<u>Statistical evaluation</u>
Threshold to perform statistical analyses on the collected material was set to 48 cases. This number was empirically identified as having adequate power while being realistic to identify in the literature. With an anticipated background risk of death after biopsy of 0,01% in general diseases, 48 patients would be needed to identify a 2% mortality risk alpha 0,05, beta 0,2 and power 0,8 (https://clincalc.com/stats/samplesize.aspx). While a 2% mortality risk would not be clinically acceptable, we anticipate a huge bias coming from that successful biopsy procedures are not being reported in the literature and thus the expected proportion of deaths in our population is expected to be higher. Correlation between patient characteristics and outcome will be analysed using logistical regression to identify markers for adverse outcome.
Regardless of the number of cases included we will describe the proportion of death, serious side effects as well as any side effect related to the biopsy with 95% confidence intervals.

References

1. Timmers, H.J., et al., *Metastases but not cardiovascular mortality reduces life expectancy following surgical resection of apparently benign pheochromocytoma.* Endocr Relat Cancer, 2008. **15**(4): p. 1127-33.
2. Khorram-Manesh, A., et al., *Mortality associated with pheochromocytoma: increased risk for additional tumors.* Ann N Y Acad Sci, 2006. **1073**: p. 444-8.
3. Brouwers, F.M., et al., *Emergencies caused by pheochromocytoma, neuroblastoma, or ganglioneuroma.* Endocrinol Metab Clin North Am, 2006. **35**(4): p. 699-724, viii.
4. Netterville, J.L., et al., *Vagal paraganglioma: a review of 46 patients treated during a 20-year period.* Arch Otolaryngol Head Neck Surg, 1998. **124**(10): p. 1133-40.
5. Amar, L., et al., *Succinate dehydrogenase B gene mutations predict survival in patients with malignant pheochromocytomas or paragangliomas.* J Clin Endocrinol Metab., 2007. **92**(10): p. 3822-8. Epub 2007 Jul 24.
6. van Hulsteijn, L.T., et al., *Quality of life is decreased in patients with paragangliomas.* Eur J Endocrinol, 2013. **168**(5): p. 689-97.
7. van Hulsteijn, L.T., et al., *No evidence for increased mortality in SDHD variant carriers compared with the general population.* Eur J Hum Genet, 2015. **23**(12): p. 1713-6.
8. Stolk, R.F., et al., *Is the excess cardiovascular morbidity in pheochromocytoma related to blood pressure or to catecholamines?* J Clin Endocrinol Metab, 2013. **98**(3): p. 1100-6.
9. Adameova, A., Y. Abdellatif, and N.S. Dhalla, *Role of the excessive amounts of circulating catecholamines and glucocorticoids in stress-induced heart disease.* Can J Physiol Pharmacol, 2009. **87**(7): p. 493-514.
10. Baraka, A., *Undiagnosed pheochromocytoma complicated with perioperative hemodynamic crisis and multiple organ failure*, in *Pheochromocytoma - A new view of the old problem.*, M. JF, Editor. 2011, InTech. p. 135-148.
11. Sauneuf, B., et al., *Pheochromocytoma Crisis in the ICU: A French Multicenter Cohort Study With Emphasis on Rescue Extracorporeal Membrane Oxygenation.* Crit Care Med, 2017. **45**(7): p. e657-e665.
12. Bancos, I., et al., *DIAGNOSIS OF ENDOCRINE DISEASE: The diagnostic performance of adrenal biopsy: a systematic review and meta-analysis.* Eur J Endocrinol, 2016. **175**(2): p. R65-80.
13. Fishbein, L., et al., *The North American Neuroendocrine Tumor Society Consensus Guidelines for Surveillance and Management of Metastatic and/or Unresectable Pheochromocytoma and Paraganglioma.* Pancreas, 2021. **50**(4): p. 469-493.
14. Lenders, J.W., et al., *Pheochromocytoma and paraganglioma: an endocrine society clinical practice guideline.* J Clin Endocrinol Metab, 2014. **99**(6): p. 1915-42.

15. Nigri, G., et al., *Meta-analysis of trials comparing laparoscopic transperitoneal and retroperitoneal adrenalectomy.* Surgery, 2013. **153**(1): p. 111-9.
16. Bravo, A.A., S.G. Sheth, and S. Chopra, *Liver biopsy.* N Engl J Med, 2001. **344**(7): p. 495-500.
17. Rockey, D.C., et al., *Liver biopsy.* Hepatology, 2009. **49**(3): p. 1017-44.
18. Tuxen, I.V., et al., *Copenhagen Prospective Personalized Oncology (CoPPO) - Clinical utility of using molecular profiling to select patients to phase 1 trials.* Clin Cancer Res, 2018. **25**(4): p. 1239-1247.
19. Page, M.J., et al., *The PRISMA 2020 statement: an updated guideline for reporting systematic reviews.* BMJ, 2021. **372**: p. n71.
