# Supplementary material for: Risk of complications after core needle biopsy in pheochromocytoma/paraganglioma
Source: Endocr Relat Cancer. 2023 Jun 2;30(7):e220354. doi: 10.1530/ERC-22-0354 (PMC10304911; doi:10.1530/ERC-22-0354)
Supplement: Supplementary Material [file supplementary_material.pdf]

Supplementary appendix

Section 1. Bias assessments

1.1. Modified Newcastle-Ottawa Scale

1.1.1. Selection bias
Low risk: Unselected case series.
High risk: All Case reports. Case series on selected materials or where patient selection is not described.

1.1.2. Baseline characteristic
Low risk: Information on the baseline data is available.
High risk: Two or more data points are not included.

1.1.3. Outcome data
Low risk: Complications of core-needle biopsy and patient survival status are fully described in the text.
High risk: Core-needle biopsy-related complications are not described.

Section 2. Descriptions of included studies

Supplementary table 1. Basic information of the included case series

**Author, year**	**Included patients**	**Gender/Age (years)**	**PPGL size(cm)**	**Catecholamine related symptoms**	**Elevated catecholamines**	**Adrenoceptor blockade before biopsy**	**Metastasis before biopsy**
Lynn M D,1987 [1]	3	F/36	NR	No	Yes	No	Yes
		M/53	NR	No	Yes	No	Yes
		M/46	NR	No	Yes	No	Yes
Stewart CJ,2002 [2]	2	NR	NR	NR	NR	NR	NR
Paulsen SD,2004 [3]	3	NR	2.5	No	No	No	NR
		NR	3.7	No	Yes	No	NR
		NR	7	No	Yes	No	NR
Weismann D,2006 [4]	1	M/50	5	No	Yes	No	No
Tsitouridis I,2008 [5]	1	NR	NR	No	No	No	NR
Yu R,2009 [6]	6	F/52	7	Yes	Avoid	No	No
		M/60	9	Yes	Avoid	No	No
		F/68	2.8	Yes	Yes	No	No
		F/69	3.5	Yes	Yes	No	No
		M/71	3.5	Yes	Yes	No	No
		F/36	4.6	Yes	Yes	No	No
Vanderveen KA,2009 [7]	8	NR	NR	NR	NR	No	No
Osman Y,2010 [8]	5	NR	NR	NR	NR	NR	No
Goers TA,2013 [9]	1	NR	NR	NR	NR	NR	No
Wang H,2014 [10]	2	NR	4.1	NR	NR	NR	No
		NR	3.2	NR	NR	NR	No
Delivanis DA,2016 [11]	7	NR	NR	NR	NR	NR	NR

Abbreviations: PPGL, pheochromocytoma/paraganglioma; M, male; NR, not reported.

Supplementary table 2. Basic information of the included case reports

Author, year	Gender/ Age (years)	PPGL size (cm)	Catecholamine related symptoms	Elevated catecholamines	Adrenoceptor blockade before biopsy	Metastasis before biopsy
Allen H A, 3rd,1988 [12]	F/20	6.5	No	Yes	NR	No
Clarnette R M,1989 [13]	M/72	NR	Yes	Yes	NR	Yes
Grabel J C,1990 [14]	F/43	7*6	No	NR	NR	Yes
Malthouse S R,1992 [15]	M/58	8	No	No	No	No
Sharma S K,1993 [16]	F/52	NR	No	NR	No	No
Takezawa Y,2001 [17]	F/66	NR	No	Yes	Yes	No
Mori S,2002 [18]	M/65	9*9*6	NR	No	NR	Yes
Yanagi Y,2002 [19]	M/59	17*15	No	No	NR	Yes
Hassan A,2003 [20]	F/24	3	No	NR	NR	No
Yashiro T,2005 [21]	F/53	NR	Yes	Yes	No	Yes
Dalal T,2005 [22]	M/75	2 * 1.5	Yes	NR	No	Yes
Sood SK,2007 [23]	M/74	7	No	Yes	No	No
	F/59	2.5	Yes	Yes	No	No
Ghayee HK,2008 [24]	F/36	3	Yes	Yes	No	No
Naito M,2009 [25]	M/59	NR	Yes	Yes	No	Yes
Mamlouk MD,2009 [26]	F/37	NR	Yes	Yes	Yes	Yes
Kunitz A,2010 [27]	M/50	24*22*12	No	NR	No	No
Fiorino KN,2011 [28]	M/10	8	No	No	No	No
Khan MR,2011 [29]	M/24	18*15	No	No	No	No
Tan M,2012 [30]	F/70	5.5	Yes	Yes	Yes	Yes
Butt N,2012 [31]	M/17	2.5*2.4	Yes	No	No	No
Padilla-Fernandez B,2012 [32]	M/34	9	Yes	Yes	No	No
Rekhi B,2013 [33]	M/54	NR	Yes	No	No	Yes

Wang HH,2013 [34]	M/53	NR	Yes	Yes	No	No
Lin Y,2013 [35]	M/69	8.7 *7.5	Yes	NR	NR	Yes
Parida GK,2014 [36]	F/75	8*8	Yes	Yes	NR	Yes
Cai T,2014 [37]	F/17	4*3.2	No	NR	No	No
Kim JK,2014 [38]	M/69	7	Yes	Yes	Yes	Yes
Sakai K,2015 [39]	M/45	25*17*15	No	No	NR	Yes
Meydan C,2016 [40]	M/49	5.9*6.4	No	Yes	No	No
Jang Khan NA,2016 [41]	M/50	NR	No	NR	NR	Yes
Xu L,2016 [42]	M/35	NR	Yes	NR	No	No
Munoz-Largacha JA,2017 [43]	F/44	5*6.4	No	Yes	No	No
Chow LTC,2018 [44]	F/34	6.8*3.7*2	Yes	Yes	No	No
Spencer D,2018 [45]	F/64	4*3	Yes	NR	No	No
Yue Y,2019 [46]	M/47	NR	No	No	No	No
Tyagi S,2019 [47]	M/76	NR	No	NR	NR	No
Fadiga L,2019 [48]	F/66	NR	Yes	No	NR	Yes
Dong ZR,2019 [49]	F/39	8*6*5	No	NR	NR	Yes
Chortis V,2019 [50]	M/65	7	Yes	No	No	No
McDermott E,2021# [51]	NR	NR	NR	NR	NR	NR
Jia Y,2021 [52]	M/64	NR	No	NR	NR	Yes
Jester G,2021 [53]	M/19	11*9	No	Yes	NR	Yes
Feng B,2021 [54]	M/48	NR	Yes	NR	NR	Yes
Jabarkheel R,2021 [55]	F/47	NR	No	NR	NR	Yes
Liu M,2022 [56]	F/67	8	Yes	NR	No	No

Abbreviations: PPGL, pheochromocytoma/paraganglioma; F, female; M, male; NR, not reported. #, this article included 2 cases.

List of included articles

1. Lynn, M.D., E.M. Braunstein, and B. Shapiro, *Pheochromocytoma presenting as musculoskeletal pain from bone metastases.* Skeletal Radiol, 1987. **16**(7): p. 552-5.
2. Stewart, C.J., J. Coldewey, and I.S. Stewart, *Comparison of fine needle aspiration cytology and needle core biopsy in the diagnosis of radiologically detected abdominal lesions.* J Clin Pathol, 2002. **55**(2): p. 93-7.
3. Paulsen, S.D., et al., *Changing role of imaging-guided percutaneous biopsy of adrenal masses: evaluation of 50 adrenal biopsies.* AJR Am J Roentgenol, 2004. **182**(4): p. 1033-7.
4. Weismann, D., et al., *A dangerous liaison--pheochromocytoma in patients with malignant disease.* Ann Surg Oncol, 2006. **13**(12): p. 1696-701.
5. Tsitouridis, I., et al., *CT guided percutaneous adrenal biopsy for lesions with equivocal findings in chemical shift MR imaging.* Hippokratia, 2008. **12**(1): p. 37-42.
6. Yu, R., et al., *Diagnosis and treatment of pheochromocytoma in an academic hospital from 1997 to 2007.* Am J Med, 2009. **122**(1): p. 85-95.
7. Vanderveen, K.A., et al., *Biopsy of pheochromocytomas and paragangliomas: potential for disaster.* Surgery, 2009. **146**(6): p. 1158-66.
8. Osman, Y., et al., *Percutaneous adrenal biopsy for indeterminate adrenal lesion: complications and diagnostic accuracy.* Urol Int, 2010. **84**(3): p. 315-8.
9. Goers, T.A., et al., *Outcomes of resection of extra-adrenal pheochromocytomas/paragangliomas in the laparoscopic era: a comparison with adrenal pheochromocytoma.* Surg Endosc, 2013. **27**(2): p. 428-33.
10. Wang, H., et al., *Ultrasound-guided core needle biopsy in diagnosis of abdominal and pelvic neoplasm in pediatric patients.* Pediatr Surg Int, 2014. **30**(1): p. 31-7.
11. Delivanis, D.A., et al., *Procedural and clinical outcomes of percutaneous adrenal biopsy in a high-risk population for adrenal malignancy.* Clin Endocrinol (Oxf), 2016. **85**(5): p. 710-716.
12. Allen, H.A., 3rd, B.W. Holmes, Jr., and J.D. Hopkins, *Bilateral ectopic pheochromocytomas: computed tomographic and sonographic correlation.* Urol Radiol, 1988. **9**(4): p. 228-30.
13. Clarnette, R.M., E.J. Bayliss, and L. Matz, *Malignant paraganglioma causing bilateral pleural effusions.* Aust N Z J Med, 1989. **19**(1): p. 61-3.
14. Grabel, J.C., et al., *Pheochromocytoma presenting as a skull metastasis with massive extracranial and intracranial extension.* Neurosurgery, 1990. **27**(1): p. 134-6; discussion 136-7.
15. Malthouse, S.R., L. Robinson, and S.C. Rankin, *Ultrasonic and computed tomographic appearances of paraganglioma simulating pancreatic mass.* Clin Radiol, 1992. **45**(4): p. 271-2.
16. Sharma, S.K., S. Sharma, and S. Mukhopadhyay, *Mediastinal paraganglioma presenting as an intracardiac mass with superior vena caval obstruction.* Thorax, 1993. **48**(11): p. 1181-2.

17. Takezawa, Y., et al., *Pheochromocytoma of the urinary bladder: a case report.* Hinyokika Kiyo, 2001. **47**(2): p. 105-7.
18. Mori, S., et al., *A case of metastatic extra-adrenal pheochromocytoma 12 years after surgery.* Hypertens Res, 2002. **25**(1): p. 141-4.
19. Yanagi, Y., et al., *Mandibular metastasis presenting as the initial maifestation of malignant pheochromocytoma.* European Journal of Radiology, 2002. **44**(1): p. 5-9.
20. Hassan, A., et al., *Paraganglioma of the vagina: report of a case, including immunohistochemical and ultrastructural findings.* Int J Gynecol Pathol, 2003. **22**(4): p. 404-6.
21. Yashiro, T., et al., *Successful surgical treatment for life threatening metastatic thoracic and cervical pheochromocytoma.* Interact Cardiovasc Thorac Surg, 2005. **4**(1): p. 75-6.
22. Dalal, T., et al., *Extraadrenal pheochromocytoma: a rare cause of tachycardia and hypertension during percutaneous biopsy.* AJR Am J Roentgenol, 2005. **185**(2): p. 554-5.
23. Sood, S.K., S.P. Balasubramanian, and B.J. Harrison, *Percutaneous biopsy of adrenal and extra-adrenal retroperitoneal lesions: beware of catecholamine secreting tumours!* Surgeon, 2007. **5**(5): p. 279-81.
24. Ghayee, H.K., et al., *The many faces of pheochromocytoma.* J Endocrinol Invest, 2008. **31**(5): p. 450-8.
25. Naito, M., et al., *R27X nonsense mutation of the SDHB gene in a patient with sporadic malignant paraganglioma.* Endocrine, 2009. **36**(1): p. 10-5.
26. Mamlouk, M.D., et al., *Radiofrequency ablation and biopsy of metastatic pheochromocytoma: emphasizing safety issues and dangers.* J Vasc Interv Radiol, 2009. **20**(5): p. 670-3.
27. Kunitz, A., et al., *Large paraganglioma of the abdominal cavity: a case report and review of the literature.* Onkologie, 2010. **33**(7): p. 377-80.
28. Fiorino, K.N., et al., *An unusual case of gastrointestinal bleeding.* Case Rep Pediatr, 2011. **2011**: p. 748543.
29. Khan, M.R., et al., *Primary non-functioning paraganglioma of liver: a rare tumour at an unusual location.* Journal of the Pakistan Medical Association, 2011. **61**(8): p. 814-816.
30. Tan, M., et al., *Malignant pheochromocytoma presenting as incapacitating bony pain.* Pain Pract, 2012. **12**(4): p. 286-9.
31. Butt, N., et al., *An incidentally discovered asymptomatic para-aortic paraganglioma with Peutz-Jeghers syndrome.* Saudi J Gastroenterol, 2012. **18**(6): p. 388-91.
32. Padilla-Fernandez, B., et al., *Paraganglioma of prostatic origin.* Clin Med Insights Case Rep, 2012. **5**: p. 69-75.
33. Rekhi, B., et al., *Metastatic paraganglioma presenting as a primary shoulder mass.* Skeletal Radiol, 2013. **42**(11): p. 1617-22.
34. Wang, H.H., et al., *Extra-adrenal paraganglioma of the prostate.* Can Urol Assoc J, 2013. **7**(5-6): p. E370-2.
35. Lin, Y., et al., *Successful treatment of paraganglioma with sorafenib: a case report and brief review of the literature.* Onco Targets Ther, 2013. **6**: p. 1559-62.
36. Parida, G.K., et al., *Pheochromocytoma presenting with remote bony recurrence twenty years after initial surgery: detection with 68Ga-DOTANOC PET/CT.* Clin Nucl Med, 2014. **39**(4): p. 365-6.

37. Cai, T., et al., *Paraganglioma of the vagina: a case report and review of the literature.* Onco Targets Ther, 2014. **7**: p. 965-8.
38. Kim, J.K., et al., *Incidentally detected inoperable malignant pheochromocytoma with hepatic metastasis treated by transcatheter arterial chemoembolization.* Endocrinol Metab (Seoul), 2014. **29**(4): p. 584-9.
39. Sakai, K., et al., *A resected case of liver metastases from extra-adrenal retroperitoneal paraganglioma with von Recklinghausen's disease 16 years after the initial surgery.* Surg Case Rep, 2015. **1**(1): p. 84.
40. Meydan, C., et al., *Takotsubo Cardiomyopathy Induced by CT-Guided Percutaneous Biopsy of a Paraganglioma.* J Vasc Interv Radiol, 2016. **27**(6): p. 926-8.
41. Jang Khan, N.A., et al., *Spinal Cord Compression By Metastatic Thoracic Spine Paraganglioma.* J Ayub Med Coll Abbottabad, 2016. **28**(3): p. 617-619.
42. Xu, L., et al., *Nocardiosis in ectopic ACTH syndrome: A case report and review of 11 cases from the literature.* Exp Ther Med, 2016. **12**(6): p. 3626-3632.
43. Munoz-Largacha, J.A., et al., *Incidental posterior mediastinal paraganglioma: The safe approach to management, case report.* Int J Surg Case Rep, 2017. **35**: p. 25-28.
44. Chow, L.T.C., M.H.M. Chan, and S.K.C. Wong, *Functional Ulnar Nerve Paraganglioma: First Documented Occurrence in the Extremity With Hitherto Undescribed Associated Extensive Glomus Cell Hyperplasia and Tumorlet Formation.* Int J Surg Pathol, 2018. **26**(1): p. 64-72.
45. Spencer, D., et al., *Unusual cardiac paraganglioma mimicking an atypical carcinoid tumor of the lung.* J Thorac Dis, 2018. **10**(1): p. E31-E37.
46. Yue, Y., et al., *Asymptomatic left posterior mediastinal functional paraganglioma: A case report.* Medicine (Baltimore), 2019. **98**(28): p. e16383.
47. Tyagi, S., et al., *Synchronous Paraganglioma Masquerading as 68Ga-PSMA PET/CT-Avid Metastasis in Carcinoma Prostate-How Specific Is 68Ga-PSMA PET/CT?* Clin Nucl Med, 2019. **44**(6): p. e420-e422.
48. Fadiga, L., et al., *Thoracic spine metastasis presenting 18 years after complete resection of a phaeochromocytoma.* BMJ Case Rep, 2019. **12**(8).
49. Dong, Z.R., et al., *Hepatic metastatic paraganglioma 12 years after retroperitoneal paraganglioma resection: a case report.* BMC Gastroenterol, 2019. **19**(1): p. 142.
50. Chortis, V., et al., *Double trouble: two cases of dual adrenal pathologies in one adrenal mass.* Endocrinol Diabetes Metab Case Rep, 2019. **2019**.
51. McDermott, E., et al., *The role of percutaneous CT-guided biopsy of an adrenal lesion in patients with known or suspected lung cancer.* Abdom Radiol (NY), 2021. **46**(3): p. 1171-1178.
52. Jia, Y., et al., *Paraganglioma with highly malignant potential involving the rib - Case report and review of the literature.* Radiol Case Rep, 2021. **16**(7): p. 1845-1850.

53. Jester, G., H. Hassanein, and A. El-Far, *Late diagnosis of metastatic pheochromocytoma in multiple endocrine neoplasia 2B with rapid clinical decline.* BMJ Case Rep, 2021. **14**(2).
54. Feng, B., et al., *18F-FDG PET/CT in a Patient With Malignant Pheochromocytoma Recurrence and Bone Metastasis After Operation-Case Report and Review of the Literature.* Front Med (Lausanne), 2021. **8**: p. 733553.
55. Jabarkheel, R., et al., *Metastatic Paraganglioma of the Spine With SDHB Mutation: Case Report and Review of the Literature.* Int J Spine Surg, 2021. **14**(s4): p. S37-S45.
56. Liu, M., et al., *A case with primary cardiac paraganglioma: imaging findings.* Radiol Case Rep, 2022. **17**(4): p. 1280-1283.
